# Metabolomics Approach for Analyzing the Effects of Exercise in Subjects with Type 1 Diabetes Mellitus

**DOI:** 10.1371/journal.pone.0040600

**Published:** 2012-07-11

**Authors:** Laura Brugnara, Maria Vinaixa, Serafín Murillo, Sara Samino, Miguel Angel Rodriguez, Antoni Beltran, Carles Lerin, Gareth Davison, Xavier Correig, Anna Novials

**Affiliations:** 1 Department of Endocrinology, Institut d’Investigacions Biomèdiques August Pi i Sunyer (IDIBAPS), Hospital Clínic de Barcelona, Barcelona, Spain; 2 Metabolomics Platform, Universitat Rovira i Virgili, Tarragona, Spain; 3 Institut d’Investigació Sanitària Pere Virgili (IISPV), Reus, Spain; 4 Sport and Exercise Sciences Research Institute, University of Ulster, Newtownabbey, Northern Ireland, United Kingdom; 5 Spanish Biomedical Research Centre in Diabetes and Associated Metabolic Disorders (CIBERDEM), Barcelona, Spain; University of Colorado Denver, United States of America

## Abstract

The beneficial effects of exercise in patients with type 1 diabetes (T1D) are not fully proven, given that it may occasionally induce acute metabolic disturbances. Indeed, the metabolic disturbances associated with sustained exercise may lead to worsening control unless great care is taken to adjust carbohydrate intake and insulin dosage. In this work, pre- and post-exercise metabolites were analyzed using a ^1^H-NMR and GC-MS untargeted metabolomics approach assayed in serum. We studied ten men with T1D and eleven controls matched for age, body mass index, body fat composition, and cardiorespiratory capacity, participated in the study. The participants performed 30 minutes of exercise on a cycle-ergometer at 80% VO_2_max. In response to exercise, both groups had increased concentrations of gluconeogenic precursors (alanine and lactate) and tricarboxylic acid cycle intermediates (citrate, malate, fumarate and succinate). The T1D group, however, showed attenuation in the response of these metabolites to exercise. Conversely to T1D, the control group also presented increases in α-ketoglutarate, alpha-ketoisocaproic acid, and lipolysis products (glycerol and oleic and linoleic acids), as well as a reduction in branched chain amino acids (valine and leucine) determinations. The T1D patients presented a blunted metabolic response to acute exercise as compared to controls. This attenuated response may interfere in the healthy performance or fitness of T1D patients, something that further studies should elucidate.

## Introduction

Type 1 diabetes mellitus (T1D) is a lifelong metabolic disorder of usual acute onset in children, adolescents and young adult people. Over time, micro and macro vascular co-morbidities develop in patients with T1D which are closely related to metabolic control [1]. In addition to these complications, the management of T1D is particularly complex and challenging. It is well known that, in order to maintain optimal glycemic control, T1D patients need accurate administration of insulin coordinated with a balanced diet and an adequate level of physical activity.

Exercise plays a crucial role in the prevention and treatment of several chronic diseases, including glucose intolerance states, type 2 diabetes [2]–[4] and diseases of the cardiovascular system [5], [6]. Moreover, it has been demonstrated that exercise improves the quality of life in the general population [7]. However, the beneficial effects of exercise in patients with T1D are not fully proven, given that exercise may occasionally induce acute metabolic disturbances, mainly related to insulin treatment. Nevertheless, children and adolescents with T1D are encouraged to exercise regularly as a means of improving social integration and cardiovascular health [8]. Thus, a better understanding of the effects of exercise on the metabolic response in T1D patients will allow clinicians to prescribe exercise to their patients with greater clarity.

Metabolomics enables the systematic assessment of the abundant changes of low molecular weight compounds present in biological samples, using high-throughput sample analysis techniques (GC-MS, NMR or HPLC-MS) and computer-assisted multivariate pattern-recognition techniques [9]. Metabolomics is enriching our current understanding of both the physiologic and physiopathologic processes underlying diabetes mellitus [10]–[12]. Moreover, recent metabolomic-based studies have described the first metabolic signatures of exercise in human plasma [13]–[15]. For example, Lewis et al [13] described the metabolic changes in tricarboxylic acid cycle, fatty acid oxidation and lypolisis in the plasma of healthy subjects exposed to different intensities and durations of exercise.

Most of the *in vivo* studies investigating the metabolic pathways of T1D have been performed under strictly controlled conditions using hyperinsulinemic euglycemic clamp techniques [16], [17] or in situations of insulin withdrawal [12]. Although these studies have provided invaluable new insights into the metabolic disturbances present in T1D, the experimental conditions used are dissimilar to everyday life. To the best of our knowledge, no study to date has applied a metabolomics approach prior to and following exercise in subjects with T1D. We hypothesize that (a) an acute bout of exercise will result in changes in the systemic metabolic profile and that (b) these parameters will be different in patients with T1D in comparison to healthy controls. The aim of this study is to analyze the metabolic changes induced by a short-term session of acute exercise performed by T1D patients and their corresponding non-diabetic counterparts. A comprehensive ^1^H-NMR and GC-MS untargeted metabolomics approach was applied to serum samples taken from all participants.

## Methods

### Participants

Ten recreationally active male patients with T1D, recruited by the Department of Endocrinology (Hospital Clinic, Barcelona), and eleven non-diabetic controls matched for sex, age, body mass index (BMI) and similar physical activity, recruited from a research institute (IDIBAPS, Barcelona), were enrolled in the study.

The T1D patients participating in the study had diabetes for a total of 14±8.4 years, undetectable C-peptide levels and good glycemic control, as determined by glycated hemoglobin A1c. Total body composition was measured by densitometry using DXA (Lunar iDXA body composition, GE Healthcare). Patients with chronic complications related to diabetes were excluded. All patients presented microalbuminuria values below 30 mg/L, normal retinal exam by direct and indirect retinoscopy, normal peripheral neurologic evaluation by clinical exploration and biothesiometry (Bio-thesiometer, Bio-Medical Instrument Company, Newbury, OH, U.S.), and normal. resting 12-lead electrocardiogram (ECG) and normal exercise testing by upright cycle-ergometer (25 W/3 min) [18]. At the time of testing, none of the T1D participants were taking any form of prescription medication, except for long-acting basal insulin analogue glargine (Sanofi-Aventis, U.S.) and fast-acting insulin analogue aspart (Novo Nordisk, Denmark).

### Experimental Procedures

All subjects were required to visit the Diabetes and Exercise Research Unit of the Hospital Clínic on two separate occasions. On the first visit, all subjects were fully briefed and familiarized with the experimental procedures. Baseline clinical characteristics such as height, weight, BMI and total and fat body composition where also obtained, and each subject was required to complete an evaluation of current physical activity using the International Physical Activity Questionnaire [19]. Maximal oxygen uptake (VO_2_max) was determined by using a maximal progressive incremental exercise test on a friction-braked cycle-ergometer (Monark 828E, Monark Sweden). After a 3 min warm-up period at a power output of 25-W, workload was increased by 25-W every minute until exhaustion. Oxygen uptake was monitored during exercise using a computerized, open circuit gas-collection system (Vmax Spectra, version v12.0, Sensor Medics Corp, VIASYS Healthcare Inc, Yorba Linda, CA, U.S.), and VO_2_max was determined at the point of highest oxygen consumption over a 15-s period. VO_2_max was confirmed using established physiological criteria, including a respiratory exchange ratio above 1.15, oxygen uptake reaching a plateau despite an increased work rate, and a heart rate near 95% of the age-predicted maximum value.

On the second visit, conducted at the same time (8:30am) a week later, subjects performed an acute bout of 30 minutes of intense exercise at 80% VO_2_max (individually calculated during the preliminary session) on a cycle-ergometer. Subjects performed 3 to 6 minutes of warm-up until achieving a fixed cardiac frequency. Prior to exercise, all participants fasted overnight for a 12 hour period, following a balanced meal consisting of approximately 55% carbohydrates, 30% fat, and 15% proteins. Furthermore, T1D patients had received their last short-acting insulin injection before dinner at 8 p.m. and their long-acting insulin injection at 10 p.m. All subjects were asked to avoid strenuous exercise and alcohol consumption the day prior to the acute exercise protocol.

#### Blood determinations

Fasting blood samples were obtained before and after the short-term intensive exercise intervention. Glycemia (glucose-oxidase method, Advia 2400 Siemens Diagnostics, Deerfield, IL, U.S.) and insulinemia (quimioluminiscent method, Siemens Healthcare Diagnostics, Tarrytown, NY, U.S.) were determined in serum samples. For the metabolomic measurements, serum was obtained once blood had been allowed to clot at room temperature for 30 min and after centrifugation at 4°C at 5000 rpm for 10 min. Samples were kept at - 80°C until further metabolomic analysis.

#### Serum ^1^H-NMR metabolomics

Serum samples were thawed, vortexed and allowed to stand for 10 min prior to NMR analysis. For NMR measurements 430 µl of serum were transferred into 5 mm NMR tubes. A double tube system was used: an internal tube (o.d. 2 mm, supported by a Teflon adapter) containing the reference substance (sodium 3-trimethylsilyl [2, 2, 3, 3-d4] propionate (TSP) 9.9 mmol/l, MnSO_4_ 0.47 mmol/l in 99.9% D_2_O) was placed coaxially into the NMR sample tube (o.d. 5 mm). This double tube system was kept at 4°C in the sample changer until analysis was performed. Spectra were acquired at a ^1^H observation frequency of 600.20 MHz at a temperature of 300 K using an Avance III-600 Bruker spectrometer equipped with an inverse TCI 5 mm cryoprobe®. The Carr-Purcell-Meiboom-Gill (cpmg, spin-spin T_2_ relaxation filter) pulse sequence with a fixed spin-spin relaxation delay of 200 ms was applied to acquire ^1^H-NMR spectra for all serum samples, in order to minimize the broad signals arising from lipoprotein and albumin in the NMR spectra. For each sample, 128 transients were collected into 32 K data points using a spectral width of 12 kHz with a relaxation delay of 2 s and an acquisition time of 1.36 s. A line-broadening function of 0.3 Hz was applied to all spectra prior to Fourier transform.

#### Serum GC-MS metabolomics

A second aliquot of serum (100µL) was used for GC-MS analysis according to Agilent’s specifications [20]. Each aliquot was spiked with 20 µl internal standard solution (1 µg.µL-1 succinic-d4 acid; Sigma-Aldrich). After protein precipitation using 900 µl of cold methanol/water (8∶1 v/v), samples were centrifuged for 10 minutes at 4°C. 200 µL of the supernatant were transferred to a GC autosampler vial and spiked with 20 µl of myristic acid-d27 (Sigma Aldrich), used as the internal standard for retention time lock (RTL system provided in Agilent’s ChemStation Software), and lyophilized overnight (Lyotrap freeze dryer). Samples were methoximated by incubating lyophilized serum residues in 50 µl of methoxyamine in pyridine (0.3 µg/µL) for 16 hours at room temperature. Silylation was subsequently done using 30 µL of N-methyl-N-trimethylsilyltrifluoroacetamide with 1%trimethylchlorosilane (MSTFA +1% TMCS, Sigma) for 1 hour at room temperature. Samples were automatically injected into a GC–MS system (HP 6890 Series gas chromatograph coupled to a mass selective detector model 5973) equipped with a J&W Scientific DB 5-MS+DG stationary phase column (30 m × 0.25 mm i.d., 0.1 µm film) (Agilent Technologies). The injector temperature was set at 250°C, and the helium carrier flow rate was kept constant at 1.1 mL/min. The column temperature was held at 60°C for 1 min, then increased to 325°C at a rate of 10°C/min and held at 325°C for 10 min. The detector operated in the electron impact ionization mode (70 eV) and mass spectra were recorded after a solvent delay of 4 min with 2.46 scans per second (mass scanning range of m/z 50–600; threshold abundance value of 50 counts). The source temperature and quadrupole temperature were 230 and 150°C, respectively.

### Ethics

Written informed consent was obtained from all subjects prior to participation. The experimental protocol was approved by the Research and Ethics committees of the Hospital Clínic de Barcelona, in accordance with the Declaration of Helsinki.

### Data Analysis and Statistical Methods

The acquired CPMG ^1^H-NMR spectra were phased, baseline-corrected and referenced to the chemical shift of the α-glucose anomeric proton doublet at 5.23 ppm. Pure standards compound reference in BBioref AMIX (Bruker) was used; HMDB and Chenomx databases were used for metabolite identification. After baseline correction, intensities of each ^1^H-NMR region identified in the CPMG 1D-NMR spectra were integrated using the AMIX 3.8 software package (Bruker, GmBH). Each region was normalized to the ERETIC (Electronic REference To access *In vivo* Concentrations) signal [21].

Raw GC/MS files were exported into the platform-independent netCDF (*.cdf) and loaded into XCMS software (version 1.6.1) based on R-program version 2.4.0 (R-Foundation for statistical computing, www.Rproject.org), where peak peaking, integration and alignment in the time domain were performed. Integrated intensities of each m/z-retention time pair (MZRT) were obtained for each one of the samples used in the study. These intensities were normalized to internal standard succinic acid-d4. The AMDIS program (Automated Mass Spectral Deconvolution and Identification System, National Institute of Standards and Technology, Gathersburg, MD, U.S.A.) was run for peak annotation, and both the Fiehn GC/MS Metabolomics RTL Library and NIST mass spectral databases were used for identification. Table S1 shows detailed identification parameters for both GC-MS and ^1^H-NMR determined metabolites.

Baseline metabolic differences between T1D and control groups were evaluated using the Mann-Witney U run test. Effects of exercise intervention on the independent control and T1D groups were assessed using the Wilcoxon exact rank sum tests. Repeated measures ANOVA were used to determine diabetes×exercise interactions. A statistically significant interaction indicates that control and T1D responded differently to the acute exercise protocol for a given metabolite. To account for multiple testing, q-values were computed for all systematic univariate tests outlined above by applying the FDR (False Discovery Rate) procedure described by Storey et al [22]. In all cases statistical significance was set at q≤0.1.Data (pre-) processing, data analysis, and statistical calculations were performed with Matlab (Matlab version 6.5.1, Release 13, The Mathworks, 2003).

## Results

### Clinical and Biochemical Baseline Characteristics

Anthropometric and fitness data are summarized in Table 1. In ten patients with T1D and eleven controls matched for age, height, weight, and body mass index (BMI), no differences were found in relation to the percentage of body fat composition and cardio respiratory capacity evaluated by VO_2_max.

**Table 1:** Clinical characteristics of T1D patients and control population.

	Control	T1D		p-values
Subjects	11	10		ns
Age (years)	32.5±8.8	35.1±8.4		ns
Evolution of diabetes (years)	–	14±8.4		–
Height (m)	1.76±0.06	1.76±0.05		ns
Weight (kg)	75.9±8.6	75.6±5.8		ns
BMI (kg/m^2^)	24.7±2.6	24.3±1.7		ns
Fat percentage (%, by DXA)	23.9±5.7	21.7±6.5		ns
IPAQ (METs min/week)	2550±995	2630±241		ns
VO_2_max (mL/kg/min)	34±9.1	35±6.5		ns
Units of insulin glargine	–	31±7.9		–

Values are reported as mean values ± SD.

ns: not significant.

At baseline (Table 2), subjects with T1D presented higher glucose and insulin levels than controls. Moreover, untargeted ^1^H-NMR and GC-MS metabolomic profiling results showed elevated levels of the tricarboxylic acid cycle intermediates (TCAIs) malate and citrate in T1D. Glycerol was also increased in the T1D group, whereas lysine levels were significantly lower as compared to the control group.

**Table 2:** Baseline analytical differences of T1D patients and control population.

	Control(n = 11)	T1D(n = 10)	q-values
**Biochemical determinations**			
Glucose (mg/dl)	90.27±2.25	202.7±24.36	5.52×10^−4^
Insulin (UI/L)	6.96±1.31	18.62±4.64	0.022
C-peptide (ng/ml)	–	undetectable	–
Glycated hemoglobin (%)	–	6.9±1	–
**Metabolomics analysis (arbitrary units)**			
Lysine	0.039±0.0036	0.025±0.0029	0.023
Glycerol	0.039±0.0022	0.045±0.0039	0.064
Citrate	0.006±0.0004	0.008±0.0005	0.052
Malate	0.001±0.0001	0.002±0.0002	0.083

Values are reported as mean values ±SEM. Selected quantitative ions relative to internal standard areas are used in the case of GC-MS measurements. Selective ^1^H-NMR regions relative to ERETIC digital signals are used in the case of NMR measurements. Two-sided p-values are calculated using Mann-Witney test. Statistical significance was set as q<0.1.

### Metabolic Changes Induced by Short-term Intensive Exercise

**Figure 1:**
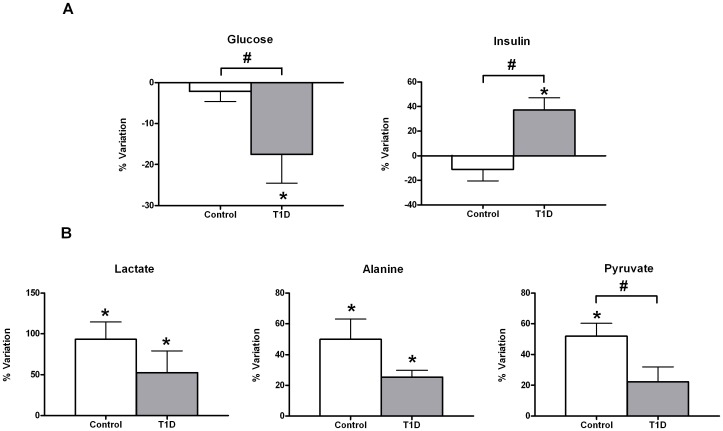
Relative changes in insulin and glucose and in gluconeogenic precursors in response to acute exercise. Relative changes in insulin and glucose (A) and in gluconeogenic precursors (B) in response to 30 minutes of acute exercise (80% VO_2_max). Percentage of variation was calculated for each individual as the levels of a certain metabolite after exercise minus the levels of the same metabolite prior to exercise relative to the former. Data are shown as mean±sem of net percent variation for T1D and control groups. A positive value of percentage of variation indicates that metabolic levels have increased in mean with exercise, whereas a negative mean denotes the opposite. *Indicates a significant variation in metabolic levels with exercise (Wilcoxon rank-summed test for the comparison of a particular metabolite level prior to and after exercise in the independent T1D and control exercisers, q<0.1). #Indicates a significant diabetes×exercise interaction for a particular metabolite (Repeated-measures ANOVA, q<0.1). Insulin and glucose data correspond to biochemical measurements, whereas lactate, alanine, and pyruvate were evaluated in ^1^H-NMR spectra according to Table S1.

Figure 1A shows a comparison of the mean net percent variation following exercise in the individual T1D and control groups for glucose and insulin. After 30 minutes of intensive exercise, glucose levels varied differently according to diabetic condition. The T1D group, hyperglycaemic at baseline, showed significant glucose depletion with acute exercise unlike their control counterparts. Insulin levels also varied differently with the exercise according to diabetic condition. While insulin levels rose significantly with exercise in the T1D group, control exercisers showed a net decrease of insulin, which did not account for statistical significance. Although after short-term acute exercise both T1D and control groups showed a significant net increase in circulating levels of gluconeogenic precursors (alanine and lactate), this increase was less pronounced in the T1D group (Figure 1B). In addition to alanine and lactate, pyruvate increased significantly with exercise in the control group but not in the T1D group.

**Figure 2:**
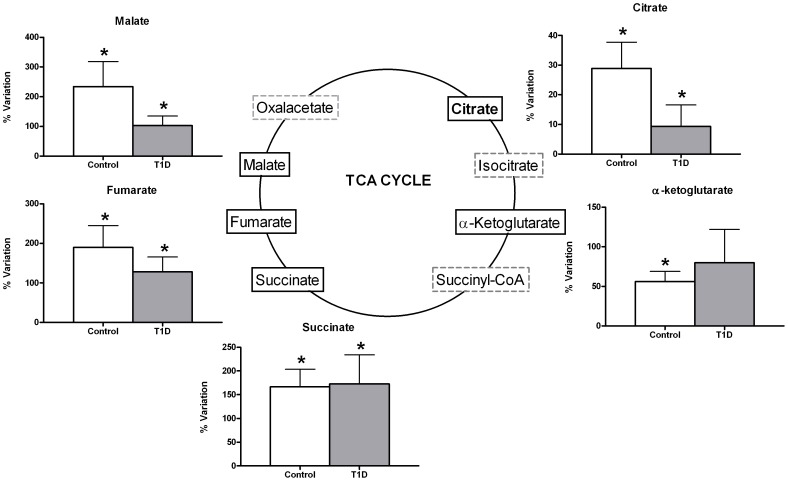
General significant enrichment in TCA cycle intermediates (TCAIs) in peripheral blood after acute exercise. Monitored using GC-MS (malate, fumarate, α-ketoglutarate) and NMR (citrate and succinate). Data are mean±sem of net percent variation with exercise. *Indicates a significant variation in metabolic levels with exercise (Wilcoxon rank-summed test for the comparison of a particular metabolite level prior to and after exercise in the independent T1D and control exercisers, q<0.1).

A significant enrichment in TCA cycle intermediates citrate, malate, fumarate, and succinate was observed after acute exercise in the peripheral blood of both control and T1D groups (Figure 2). However, α-ketoglutarate levels significantly increased only in the case of control exercisers. In general terms, there was less accumulation of TCA cycle intermediates in serum in the T1D group. No changes in other TCAIs were identified (isocitrate, succinil-coA and oxalacetate), nor was any interaction observed between the groups of T1D and controls in the metabolites that increased following exercise.

**Figure 3:**
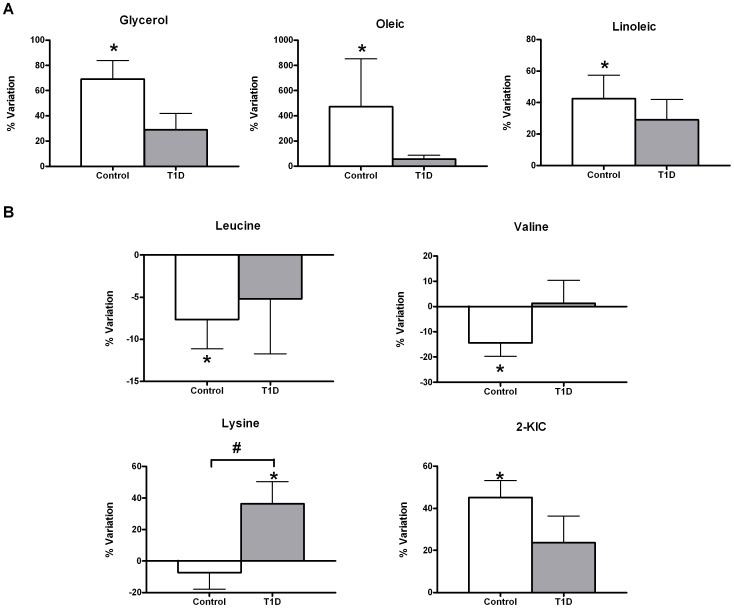
Relative changes in lypolysis (A) and BCAA metabolism (B) with acute exercise. Data are mean±sem of net percent variation. *Indicates a significant variation in metabolic levels with exercise (Wilcoxon rank-summed test for the comparison of a particular metabolite level prior to and after exercise in the independent T1D and control exercisers, q<0.1). #Indicates a significant diabetes×exercise interaction for a particular metabolite (Repeated-measures ANOVA, q<0.1).

Exercise significantly increased glycerol and oleic and linoleic acid concentrations in the control group exclusively. This effect was attenuated in the T1D group (Figure 3A). There was no interactive effect observed between the T1D and control groups in these products of lipolysis. Branched chain amino acids (valine and leucine) were lower following short-term acute exercise in the control group (Figure 3B). These changes were paralleled by significantly increased levels of alpha-ketoisocaproic acid (2-KIC) in the same group. This increase was shown to be a diabetes-dependent trend (p-interaction diabetes×exercise = 0.05) but not statistically significant after FDR correction at the established conventional significance level (q≤0.1). Worth mentioning is lysine, which also showed a diabetes-dependent effect with the exercise.

Metabolite identification parameters are presented in supporting table (Table S1).

## Discussion

The principal aim of this study was to analyze the metabolomic profile at rest and after a short period of intense exercise in patients affected by T1D to provide a comprehensive insight into the physiological effects of exercise on this particular population. Based on serum sample analysis, we have compared the metabolic response to short term acute exercise in T1D and healthy subjects using an untargeted metabolomics approach (GC-MS and ^1^H-NMR). Our findings revealed similar metabolic events in T1D patients and their control matched exercisers, although the T1D patients showed an attenuation of overall metabolic response after intense short-term exercise.

As it is commonly known, in order to increase energy supply during intense short-term exercise, glycogen breakdown is induced to provide the substrate for activating anaerobic glycolysis, resulting in the accumulation of pyruvate and lactate in plasma. Recently, Lewis et al [13] confirmed these results by using LC-MS-based metabolomics in a non-diabetic population. Our data, using a different metabolomic approach based on ^1^H-NMR and GC-MS techniques, showed that the non-diabetic group presented an increase in serum lactate and pyruvate concentration after exercise, indicating that glycolysis was activated, as previously reported by other groups [13], [23]. However, the T1D patients elicited only an increase in lactate levels having blunted the pyruvate response, suggesting that exercise induces a reduced activation of glycogenolysis and glycolysis in the T1D group as compared to the control group. Given the inhibitory effect of insulin on glycogen breakdown, the higher insulin levels observed in the T1D group could explain in part the attenuated glycogenolytic response.

Our analysis demonstrated a significant increase in TCAIs (malate, citrate, succinate, fumarate and α-ketoglutarate) in the control subjects in response to exercise. These results are in accordance with a previous metabolomics-based study investigating the plasma signature of exercise [13], in which accumulated levels of malate, fumarate and succinate were reported following 60 minutes of exercise in a healthy cohort. Other studies have demonstrated that an increase in the total concentration of TCAIs is necessary to enhance and maintain TCA cycle flux during strenuous exercise [24]. In addition, a positive correlation has been demonstrated between the total concentration of TCAIs and the estimated TCA cycle flux in human skeletal muscle taken from muscle biopsies during exercise [25]. In the case of patients with T1D, our results also pointed to a less significant accumulation of TCAIs after exercise, which might also compromise the TCA flux rates. Given that many of the reactions that lead to a net influx of TCAIs are directly or indirectly dependent on the level of pyruvate and that elevated concentrations of pyruvate appear to be necessary for the anaplerosis pathway [25], we suggest that the insufficient increase in pyruvate concentrations in T1D subjects might have an impact on TCA cycle replenishment. In this sense, our findings of an attenuated enrichment of TCAIs in the serum of T1D patients in response to a short period of intense exercise indicate that the activation of the TCA cycle flux rates might be affected. This suggests that the T1D group has a compromised oxidative aerobic system as compared to the control group, as the T1D group did not show a significant turnover of TCA metabolites.

Concerning lipolysis, our data demonstrated that the healthy controls showed an increase in free fatty acids and glycerol in response to exercise, however, this response was attenuated in the T1D group. The increase in lipolysis after acute exercise had been previously reported in healthy individuals [13], [26]. Under conditions of hyperinsulinemia induced by clamp techniques, a suppression of the intramuscular and subcutaneous adipose tissue lipolysis was demonstrated in healthy volunteers [27], [28]. In another study performed in obese men, a delay in the lipolytic activation was observed together with an increase in plasma insulin levels following 30 minutes of acute resistance exercise, a finding that the authors attributed to an increase in insulin levels [26]. The increased insulin levels found in the T1D group in our study could have a role in the attenuated lipolytic action observed following exercise.

Our results demonstrated that control exercisers presented a significant reduction in leucine levels. It is well established that exercise increases energy expenditure, resulting in the promotion of amino acid catabolism in general and, in particular, in the oxidation of branched chain amino acids, mainly leucine [13], [29]. In parallel to the reduction of leucine, we observed an increase in circulating levels of 2-KIC, which is the first step in leucine degradation. This increase in 2-KIC is in concordance with a previous reported study on the metabolomic profiling performed on urine samples of men following acute exercise [15]. Our results suggest that the effect induced by exercise on protein catabolism was attenuated in the diabetic patients, in agreement with other authors who have previously demonstrated a decrease in protein catabolism in T1D patients [17]. Moreover, there are evidences confirming that elevated insulin levels induced by infusion in healthy men promote muscle protein anabolism by inhibiting protein breakdown [30] and elicit the ability to stimulate glucose uptake and alanine transport, thus suppressing protein degradation in skeletal muscle [31]. Taking all these data into consideration, we propose that the high insulinemia levels induced by exogenous insulin administration in our T1D group could be the cause of reduced protein breakdown, as demonstrated by the minor alterations observed in the levels of leucine and 2-KIC following exercise.

Of special note, we detected an increase in insulin serum levels in all the T1D patients after 30-minute of acute exercise. This increase may explain in part the attenuation in the metabolomic response of all energetic substrates. Pharmacokinetic studies have shown that the peak action of insulin glargine is usually within the first 3 or 4 hours after injection [32], and in our protocol, samples were taken 10 hours later. One possible explanation for the increased insulin concentration detected in the serum of our T1D group could be that intense exercise induces the lipolysis of subcutaneous adipose tissue where insulin is stored and rapidly released into the circulation. In line with this hypothesis, Davison et al [33] previously reported an exercise-induced lipolysis effect by monitoring the release of liposoluble vitamins from the subcutaneous tissue into the bloodstream, an effect which may be responsible for the increased insulin levels observed in our T1D patients.

Although we speculate that high insulin levels induced by exogenous treatment may be responsible for the attenuated response of metabolites to acute exercise, alternative explanations must also be considered. For example, the possible presence of insulin resistance (IR), an important condition described in T1D patients, cannot be ignored. Some authors have considered that supra-physiologic levels of exogenous insulin [34] and hyperglycemia per se [35] could be responsible for IR in T1D patients. Insulin, not the rate of glucose disposal per se, regulates glycogen synthesis, meaning that the low level of glycogen synthase activity found in insulin-resistant states is a consequence of impaired insulin action, rather than reduced glucose disposal. Nevertheless, this assumption has been challenged in a more recent study in which adult patients with T1D exhibited both impaired glucose utilization and impaired insulin-induced non-esterified fatty acid suppression [36]. In addition, these patients showed IR in hepatic and skeletal muscle tissue, despite good glycemic control [37]. Another report [38] demonstrated that T1D adolescents had significantly impaired functional exercise capacity and decreased insulin sensitivity as compared to non-diabetic adolescents. In spite of their IR and reduced cardiovascular fitness, T1D youth showed paradoxically normal intramyocellular lipid content (IMCL). This finding contradicts the previously well- established theory that IMCL accumulation is a marker for insulin resistance in both T1D and T2D [39], [40].

Metabolic flexibility defined as the ability to switch from fat to carbohydrate oxidation is usually impaired during a hyperinsulinemic clamp in insulin-resistant subjects. Thus, the phenomena of metabolic inflexibility mainly described in T2D and other insulin-resistant states could explain some of the alterations occurring in the machinery of lipid and glucose metabolism [41], [42]. In addition, the inability to modify fuel oxidation in response to changes in nutrient availability has been implicated in the accumulation of intramyocellular lipids as well as in insulin resistance. Exercise represents a paradigm which requires a highly regulated coordination between fuel supply and oxidative machinery. The assumption that metabolic inflexibility may affect energy metabolism under exercise conditions in T1D patients remains to be elucidated.

Several studies involving T2D patients and their offspring have demonstrated the presence of mitochondrial dysfunction [43], [44]. In T1D patients, mitochondrial dysfunction has also been described, contributing to abnormalities in the TCA cycle and fatty acid metabolism. A recent report [45] verified that the mitochondrial capacity of untrained women with T1D correlates positively with glycemic control. The hypothesis that mitochondrial abnormalities may be a primary cause of metabolic inflexibility and insulin resistance has been offered. Significant differences in mitochondrial number, structure and function have been described between insulin-resistant and insulin-sensitive subjects, but the causal link still remains unknown [46].

Although our study was not designed to analyze IR or metabolic flexibility, these factors might have influenced the metabolomic spectrum described in our T1D patients. In addition, the presence of hyperglucagonemia and impaired glucagon counterregulation, as reported in several studies [47], could affect lipolysis, gluconeogenesis and protein metabolism. Finally, other situations not present in our patients should be taken into account, such as autonomic dysfunction, which influences lypolitic responsiveness [48], cardiac dysfunction, which has been documented in T1D patients as affecting exercise responses [49], [50], and abnormal blood flow during exercise, which could affect muscular vascular function, contributing toward metabolic disturbances [51].

In summary, we report that T1D patients have an attenuated metabolic response as compared to their healthy control counterparts after a short period of acute, intense exercise. We speculate that exercise could mobilize the subcutaneous exogenous insulin depot in adipose tissue. Furthermore, our data suggest that high insulinemia levels might play a role in the attenuated response in lipolysis, proteolysis, glycogenolysis, and oxidative metabolism observed in T1D patients following exercise. Whether the attenuation of metabolic response to acute and intense exercise might interfere in the training performance of T1D patients remains to be elucidated, and additional studies are required.

## Supporting Information

Table S1 **Metabolite identification parameters. ^1^H-NMR**: proton nuclear magnetic resonance spectroscopy; **δ**: chemical shift; **GC-MS**: gas chromatography-mass spectrometry; **RT**: retention time
(DOC)
