# Supplementary material for: Metabolomics Approach for Analyzing the Effects of Exercise in Subjects with Type 1 Diabetes Mellitus
Source: PLoS One. 2012 Jul 11;7(7):e40600. doi: 10.1371/journal.pone.0040600 (PMC3394718; doi:10.1371/journal.pone.0040600)
Supplement: Table S1 — Metabolite identification parameters. 1H-NMR: proton nuclear magnetic resonance spectroscopy; δ: chemical shift; GC-MS: gas chromatography-mass spectrometry; RT: retention time (DOC) [file pone.0040600.s001.doc]

**Table S1. Metabolite identification parameters**

| **^1^H-NMR Metabolites** | **δ(ppm)** | **multiplicity** | **Moieties** | |
| --- | --- | --- | --- | --- |
| Glycerol | 3.65 | double doublet | Half (-CH_2_-) | |
| Citrate | 2.52 | doublet | Half (-CH_2_-) | |
| Lactate | 1.33 | doublet | -CH_3_ | |
| Succinate | 2.39 | singlet | (-CH_2_-) | |
| Pyruvate | 2.36 | singlet | (-CH_2_-) | |
| Alanine | 1.46 | doublet | -CH_3_ | |
|  |  |  |  | |
| **GC-MS Metabolites** | **RT(min)** | **Quantitative ion (m/z)** | |  |
| Malate | 12.87 | 245 |  | |
| Lysine | 17.02 | 200 |  | |
| Fumarate | 11.0 | 245 |  | |
| α-ketoglutarate | 13.88 | 198 |  | |
| Oleic acid | 20.48 | 339 |  | |
| Linoleic acid | 20.43 | 337 |  | |
| α-ketoisocaproic acid (2-KIC) | 9.13 | 200 |  | |
| Leucine | 8.33 | 170 |  | |
| Valine | 9.22 | 144 |  | |
